# Consumers’ response to genetically modified food: an Italian case study

**DOI:** 10.1080/21645698.2024.2417473

**Published:** 2024-11-06

**Authors:** Federica DeMaria, Simona Romeo Lironcurti, Federica Morandi, Alessandra Pesce, Laura Gazza, Francesca Nocente

**Affiliations:** a CREA – PB Research Centre for Policies and Bioeconomy, Rome, Italy; b CREA – IT Research Centre for Engineering and Agro-food Processing, Rome, Italy

**Keywords:** Biotechnologies, consumer attitude, consumer knowledge, genetic modification, multinomial logit model

## Abstract

The agricultural sector could benefit from biotechnologies in addressing challenges such as pests, droughts, and food supply issues. Genetically modified (GM) crops have been developed to offer not only economic advantages to farmers but also to contribute positively to the environment, human health, and consumer well-being. However, consumers’ hesitancy in buying GM food may stem from societal reactions to how biotechnologies in agriculture have been regulated so far. The legislative debate that led, in early 2024, to the approval of Commission’s proposal (COM(2023) 411 final) – aimed at simplifying the authorization process for plants obtained with certain new genomic techniques (NGTs) – has sparkled public discussion in the European Union on the application of biotechnologies in agriculture. This work aims to investigate Italian consumers’ acceptance toward GM food. Through data collected from an original survey (*N* = 564), we tested a) their level of knowledge of GM techniques; b) if they are aware of differences between established techniques based on classical approaches of crossing and selection and more precise biotechnological techniques; c) their propensity to buy GM food, with a specific focus on food safety and environmental sustainability issues. By using a Multinomial Logit Model (MNL), starting from three hypotheses, the study highlights a gap in knowledge transfer and, in general, the communication process. This results in widespread misinformation that hinders informed consumer choices. The study also emphasizes consumers’ sensitivity to food safety, including environmental issues, but still related to food safety issues.

## 1. Introduction

The world population growth expected in the coming years will inevitably lead to an increase in food demand, which is forecast to rise between 35% and 56% between 2010 and 2050.^1^ Consequently, an intensification of the pressure on natural resources will occur. These aspects are closely linked to climate change, which impacts agriculture and the sustainability of global food systems.^2,3^ Using Genetic Modification (GM) techniques can potentially improve production and product quality, and promote environmental sustainability. This can be achieved by creating opportunities for both agriculture and the environment, and by improving the performance of the agricultural and food sector.^4–8^

Over the past two decades, the use of biotechnology in the agricultural sector has significantly reduced the use of plant protection chemicals by 37%, and pesticides by 8.2%. This has led to a 22% increase in yield and reduced costs)^9,10^. One of the most striking and achievable examples is linked to improving the environmental sustainability profiles of the traditional varieties of Italian viticulture, reducing the use of pesticides without altering the organoleptic characteristics of the wines. Even in the zootechnical sector, interesting perspectives on animal welfare and the enhancement of dairy could be developed. These applications give biotechnologies the potential to enhance innovation and sustainability in the European agri-food system, contributing to the achievement of the objectives of the *European Green Deal*^11^ and the *Farm to Fork* Strategy.^12^ ^a^^a.^«The European Green Deal is a package of policy initiatives, which aims to set the EU on the path to a green transition, with the ultimate goal of reaching climate neutrality by 2050. It supports the transformation of the EU into a fair and prosperous society with a modern and competitive economy. It underlines the need for a holistic and cross-sectoral approach in which all relevant policy areas contribute to the ultimate climate-related goal. The package includes initiatives covering the climate, the environment, energy, transport, industry, agriculture and sustainable finance – all of which are strongly interlinked».^11^ https://www.consilium.europa.eu/en/policies/green-deal/. Among the sectoral strategies developed under the *Green Deal*, the one specifically concerning the agri-food sector is the *Farm to Fork Strategy*, which sets the following objectives for 2030:− 50% reduction in the use of chemical pesticides, with particular focus on reducing the use of more hazardous pesticides− 50% reduction in nutrient losses, while ensuring no deterioration in soil fertility.− 20% reduction in the use of fertilizers.− 50% reduction in the sales of antimicrobials used for farmed animals and in aquaculture.− Achieve 25% of agricultural land under organic farming.− Promote sustainable food consumption and facilitate the shift to healthy, sustainable diets.− Combat food waste, aiming to reduce food waste at both the retail and consumer levels by 50%. reduction

The authorization, supervision, and labeling of GMOs and GM products within the EU are governed by Directive 2001/18/EC. However, recent advancements in genetic engineering have sparked public debate in the EU, prompting a revision of the current regulatory framework. At the end of 2019, the Council of the European Union requested the European Commission to conduct a study to clarify the implications of New Genomic Techniques (NGTs),^13^ which encompass a wide range of techniques, many of which allow for genome corrections without requiring DNA manipulations or changes to the plant’s genetic background. Consequently, organisms produced through these NGTs can be nearly identical, or even indistinguishable, from those produced through conventional breeding. The study concluded that the existing GMO regulations in the EU do not suit plants produced with these types of NGTs, as the authorization procedures and assessment criteria for GMOs are considered both disproportionate. Based on the study’s findings, the European Commission introduced a new regulation in July 2023 regarding plants developed using certain NGTs (COM(2023) 411 final), approved by the European Parliament in February 2024. The regulation proposes exempting Category 1 NGT plants – those containing genetic material from the same species (targeted mutagenesis) or from crossable species (cisgenesis, including intragenesis) – from the current GMO regulations. In contrast, transgenic plants that contain genetic material from non-crossable species will continue to be regulated under the existing GMO legislation.^14^

Public mistrust of the use of biotechnologies in agriculture could arise from societal reactions to how GM foods have been regulated so far.^15^ Scholars agree that consumers’ perceptions are heavily influenced by government decisions to approve or ban GM crop cultivation.^16–20^ A recent study by^21^ stresses the need for collaboration between life sciences and social sciences in encouraging consumer acceptance, by highlighting the importance of scientific evidence on the use of NGTs. Consumers who strongly oppose the use of NGTs will not be swayed by benefits alone; they require solid scientific evidence to outweigh potential risks. The social stigma toward GM foods, such as neophobia and health risks posed by innovative technologies, often shapes consumers’ perception of these products. A high education level could even lead to overestimating consumers’ actual knowledge level, resulting in a lower acceptance of GM foods.^9^

As regards the role of information in consumers’ acceptance, the literature is still controversial. Some scholars strongly argue that promoting the benefits of GM foods for environmental sustainability and food security may increase acceptance);^22,23^ others, on the contrary, regard the role of information as ineffective,^24^ especially when the level of information does not consider the socio-demographic characteristics of the consumer. Although there is plenty of information available for consumers to assess the risks and make informed choices, consumers still lack awareness about the pros and cons of GM food. The cause may be due to the existence of a certain disparity between consumers’ objective and subjective knowledge, which affects their attitudes toward purchasing GM foods.^25^ However, enhanced educational levels can help to better understand product labels and make purchasing decisions that align with their values. It is essential to conduct further research on the risks and benefits of GM food production to provide consumers with credible and reliable information and based on empirical evidence.^26^

Research evidence from^27^ suggests that benefit perception plays a substantial role in accepting NGT applications. Moreover, scholars agree that the acceptance of GM foods is probably affected by the specific values of some people groups, including overall concerns about global food and food security, climate change, and ethical beliefs.^26^

The scope of our study is to evaluate consumers’ attitudes toward GM foods and crops, both concerning food safety and environmental impact. Using data from an original survey conducted in 2023, we analyzed the consumers’ answers relating to knowledge of GM techniques (including NGTs, sensitivity toward environmental sustainability and food safety, and the role of information tools. Using a Multinomial Logit Model (MNL), we studied consumer preferences for GM products, evaluating the impact of various factors on purchasing decisions.

We believe that the factors driving their choices are mainly related to their knowledge level and informational tools. The trade-off between objective and subjective knowledge highlights the consumers’ level of misinformation (bad or distorted information), which may lead to an overestimation of their knowledge and, therefore, a lack of awareness and coherence in their choices. Finally, we investigate the sensitivity related to the issues of environmental sustainability and food safety.

To build our analysis, we test three hypotheses concerning the knowledge of GM techniques, environmental and food safety perceptions of food products derived from GM techniques, and the quality of information on the topic.

The test of the first hypothesis aims to assess the consumer’s level of knowledge regarding GM techniques, both in terms of their subjective perception of knowledge and objective understanding. However, our study faces a limitation related to constructing the objective knowledge variable, which relies on production-based knowledge and might not correctly reflect the level of information available to consumers.

H1: Objective and subjective knowledge of consumers about GM techniques may not always be aligned.

In the second hypothesis, we are testing consumers’ perception of environmental sustainability and food safety of products derived from GM techniques. Specifically, we expect a heightened sensitivity among consumers toward food safety issues, although we acknowledge that the environmental component also carries considerable weight.

H2: Consumers’ sensitivity toward the use of GM techniques in agriculture varies depending on whether environmental sustainability or food safety issues are considered.

Through our third hypothesis, we aim to test how consumers use information tools and how the latter influence their choices.

H3: The level of information available is significantly lacking and distorted, primarily accessible through nonscientific channels such as social media and the internet.

## 2. Materials and Methods

### 2.1. Survey Analysis Design

A survey analysis was conducted to reveal Italian consumers’ knowledge level of biotechnologies and their purchasing/consumption attitudes toward GM foods. The questionnaire is composed of four sections, and participants were asked to select between multiple-choice options. The first section includes a self-assessment of consumers’ knowledge, the second explores tools used to collect information on GMOs, the third regards issues related to the environment and food safety, and, finally, questions about the propensity to purchase GM products. The sample was stratified by gender and age to capture a larger number of consistent answers in maximizing efficiency, while income, education, household, and region were used as control variables.

### 2.2. Data Collection

To address our research question, we collected original data from a sample of 564 males and females living in Italy, who responded to 15 questions presented in Italian. The survey was conducted in January 2023 by the Appinio research agency (Appinio DEU, Hamburg, Germany) utilizing a Computer-Assisted Web Interviewing (CAWI) methodology. This approach involved distributing the survey online via e-mail and the Appinio application to a pool of respondents selected through random probability sampling. The questionnaire was programmed on the Appinio platform and underwent testing by both the Appinio Research team and the CREA team using a test link provided by Appinio. No modifications were made following the testing phase.

The study was conducted on a national level, involving individuals aged between 18 and 65 years old, and the sample followed a national representative distribution, based on the latest (2023) census data provided by ISTAT (The Italian National Institute of Statistics). More specifically, it was built to represent the Italian population by age, gender, and regional geographic (Tables 1 and 2):

1. The average deviation of the collected sample from the official population in terms of geographic distribution is of 0,0%.
2. The average deviation of the collected sample from the official population in terms of age and gender distribution is of 0,02%.

**Table 1.** Sample distribution by age and gender (N = 564).

ISTAT (as of January 2023)			Appinio Study		
	Total men	Total women		Total men	Total women
**18-24**	5.9%	5.4%	**18-24**	5.3%	5.3%
**25-34**	8.8%	8.3%	**25-34**	8.3%	9.6%
**35-44**	9.8%	9.7%	**35-44**	10.6%	11.2%
**45-54**	12.6%	12.8%	**45-54**	13.5%	12.6%
**55-65**	13.0%	13.7%	**55-65**	11.0%	12.4%

**Table 2.** Sample by geographic distribution (N = 564).

	ISTAT (as of January 2023)	Appinio Study
Piedmont	7.2%	7.1%
Aosta Valley	0.2%	0.2%
Lombardy	16.9%	19.1%
Trentino-South Tyrol	1.8%	1.6%
Veneto	8.2%	8.2%
Friuli-Venezia Giulia	2.0%	2.3%
Liguria	2.6%	2.8%
Emilia-Romagna	7.5%	6.2%
Tuscany	6.2%	4.4%
Umbria	1.5%	1.1%
Marche	2.5%	2.0%
Lazio	9.7%	10.3%
Abruzzo	2.2%	2.8%
Molise	0.5%	0.9%
Campania	9.5%	9.6%
Apulia	6.6%	6.7%
Basilicata	0.9%	1.1%
Calabria	3.1%	2.5%
Sicily	8.2%	7.8%
Sardinia	2.7%	3.4%

All the additional socio-demographic data, on education level and personal annual income, were collected on a natural fallout basis (Table 3).

**Table 3.** Demographic characteristics (N = 564).

	N	(%)
**Gender**		
Male	275	(48.8)
Female	288	(51.1)
No response	1	(0.2)
**Income**		
€0-€15,000	160	(28.4)
€15,000-€30,000	249	(44.3)
€30,000-€50,000	99	(17.6)
€50,000+	55	(9.8)
**Education**		
Doctoral Degree	27	(4.8)
Master’s degree	98	(17.4)
Bachelor’s degree	77	(13.7)
High school diploma	304	(53.9)
Middle school diploma	55	(9.8)
No qualification	3	(0.5)
**Household**		
1	85	(15.1)
2	158	(28.0)
3	175	(31.0)
4	115	(20.4)
5	27	(4.8)
6+	3	(0.5)
No response	1	(0.2)

The data collected is compiled and presented anonymously in a statistical evaluation that prevents any identification of individual users.

Details concerning the specific data usage practices of Appinio (ethical approval) can be found in Appendix A.

**Figure 1.**
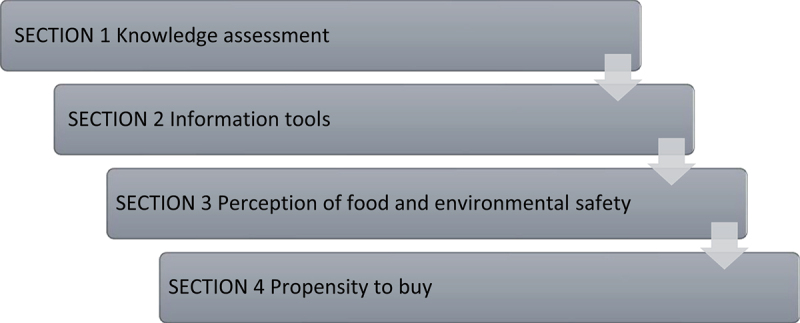
Survey Structure.

In the first part of the survey, as shown in Figure 1, respondents answered four questions about GM foods. Three of these are related to subjective opinions and one is related to objective facts. Among subjective opinions, a question is related to what genetic improvement methods are known (multiple choice), mainly distinguishing between techniques that are used regardless of genetic modification and new techniques, such as GMOs or NGTs. The second and third questions investigate the differences between NGTs and GMOs and the purpose of genetic modification. The last question is related to objective knowledge and investigates the most cultivated agricultural species as GM seeds.

Section two includes details on the use of tools used to obtain information on GM products. The variable related to information sources is coded as a sum of the total number of tools used by consumers. Section three explores consumer perceptions of GM foods, with a focus on safety concerns related to health and the environment. The last section of the survey aims to investigate the consumers’ perspective on buying GM products, and the propensity to purchase GM foods. Furthermore, the propensity to buy depending on the product’s origin is also investigated.

Results of the answers highlight that the most known genetic improvement methods are GMOs (68%) and in vitro culture techniques (47%). About 57% of the sample say there are differences between GMO techniques and new breeding techniques (NBTs), but only 15% understand these differences. Concerning food safety, 34% of Italians consider GM foods safe to eat. A study by Wolf et al.^28^ confirms that Italians maintain a certain level of skepticism toward GM food; however, we find that opinions regarding the potential safety of GM crops for the environment have improved since then. Of the sample, 38% believe GM productions are fairly or very safe, while 55% of the sample believe GM productions can promote environmental sustainability. About the scope of genetic modifications, resistance to insects, diseases, and herbicides is recognized as the main purpose (48%), followed by adaptation to climate change. Fewer than half of respondents (33%) use scientific publications for self-knowledge assessment. The most commonly used tools for information dissemination are the press, television, and social media (84%), which could play a negative role in the acceptance of genetic improvement techniques.^29^ Finally, 4 out of 10 Italians would buy a GM product coming from Western Europe (61%), followed by North America origin (49%). Respondents display lower confidence for African (21%) and Asian (24%) products. We tested for the correlations between objective and subjective knowledge with the educational level and results indicate that consumers with a high school diploma tend to admit uncertainty in their knowledge of GMs. Further details on correlations, together with a full version of the questionnaire and an in-depth analysis of the survey results, are available in Romeo Lironcurti S. et al.^30^

### 2.3. Econometric Model

In the quantitative section, we study the predisposition to purchase products deriving from genome editing and whether and how much consumers know the techniques of genetic modification, for instance. In modeling the attitude to purchase GM products we use a Multinomial Logit Model (MNL); we include in the equation variables related to the information tools used and indicators that consider environmental sustainability and food safety issues with the product’s origin.

The Logit model for multiple-choice problems takes the following form:

(1) $$\mathrm{Pr}\left\{Y_{i}=j\right\}=\frac{\exp\left(x_{i}\beta_{j}\right)}{1+\exp\left\{x_{i}\beta_{2}\right\}+\exp\left\{x_{i}\beta_{3}\right\}+\ldots +\exp\left\{x_{i}\beta_{M}\right\}};\text{\textbackslash{}breakj}=1,2\ldots M$$

Where x_i_ is a K-dimensional vector containing the characteristics of individual *i* (including an intercept term) and *β*_*j*_ denotes a vector of alternative-specific coefficients. We estimate K-1 slope coefficients plus an intercept term for all but one of the alternatives.

The marginal effects, that is the effect of changing a regressor by one unit on the probabilities of choosing each alternative, are:

(2) $$\frac{\partial \mathrm{Pr}\left(Y_{i}=j\right)}{\partial x_{i}}=\mathrm{Pr}\left(Y_{i}=j|x\right)\left({\hat{\beta }}_{\mathrm{jk}}-\overset{M}{\underset{j=1}{\sum }}{\hat{\beta }}_{\mathrm{jk}}*\mathrm{Pr}(Y_{i}=j|x)\right)$$

Where the term $\left({\hat{\beta }}_{\mathrm{jk}}-\sum_{j=1}^{M}{\hat{\beta }}_{\mathrm{jk}}*\mathrm{Pr}(Y_{i}=j|x)\right)$ is the marginal effect and its sign may or may not correspond to the sign of the estimated coefficient. Several robustness tests will be used to validate the estimations results.

Based on the questionnaire response we have generated several variables by transforming some of the qualitative responses into quantitative ones (level of objective knowledge, and propensity to buy products from the main world blocs). The variables that capture the propensity to purchase GM products and the level of presumed knowledge are based on a Likert scale.

The multinomial regression examines the determinants influencing consumers’ likelihood of buying GM products. Given that the dependent variable is not specified in any order of importance or magnitude, this study used an unordered MNL.

The dependent variable is a discrete variable ranging between 0 and 2, where 0 indicates “Not at all,” 1 indicates “I do not know,” and 2 indicates “Most of it.” Specifically, the independent variables included in the regression model as determinants are gender, age, education, income, perception of food safety and environmental sustainability, media tools used for searching for information regarding GM products, and the product’s origin.

Table 4 provides a description of the variables used in the regression analysis.

**Table 4.** Variables description.

Variable name	Description	Details
w_buyGMpr	The dependent variable ranges between 1 and 3.	Propensity to purchase GM products.
Food_sust	A score ranging between 0 and 3 (0 = unsafe; 2 safe)	Consumers’ perception of Food GM sustainability
Env_sust	A score ranging between 0 and 2. (0 = unsafe and risky; 2 safe and no risky)	Consumers’ perception of Environmental GM sustainability
Sub_Knl	Score on Linkert scale (1= None at all; 5= Most of it)	Level of presumed GM knowledge
Total_benefit	A score ranging between 0 and 4 (0=unsafe; 4 safe and no risk)	Level of total benefit as sum between environmental sustainability and food sustainability
ObJ_Knl	Scores ranging from 1 to 5	Level of objective knowledge
I_tools	Scores ranging from 1 to 4	Number of Information Tools Used
Product_origin	4 Dummy variables if the origin of the product is America or Europe, or Asian o Africa	Product of origin
Gender	Dummy variable: 0 if male, 1 if female	Control variable
Areas/regions	Score = 1 if North-east; = 2 if North-west; = 3 if centre, = 4 if South	Control variable
Age	Age of respondents ranges between 18–65. 5 classes 1 = 18 < age < 27 2 = 28 < age < 40 3 = 41 < age < 50 4 = 51 < age < 60 5 = > 60	Control variable
Family_m	Number of family members	Control variable number of family members
Income	Scores ranging from 1 to 4 1 < 15000 euros 2 = 15000 & ≤ 30000 3 > 30000 & ≤ 50000 4 > 50000	Control variable level of income
Marital Status	Dummy Variables 0= single; 1=in a relationship	Control variable
Degree	Different classes ranging from 1 (=no degree) to 5 (=PhD):	Control variable

## 3. Empirical Results

We estimate several MNLs and differentiate the models through the inclusion each time of different variables concerning food safety, environmental sustainability, and total benefit resulting from the sum of both. Beta coefficient results are reported in the Appendix C (Tables C1, C2, and C3). We use the Akaike test (AIC) to choose which model better fits the data.

In our regression analyses, we did not account for the effects of age groups and educational level, variables that potentially hold significance in determining consumer purchasing decisions. Therefore, we deepen our analysis by incorporating these new variables into our estimations (Tables C2 and C3). Notably, both age and educational level consistently demonstrate statistical significance, thereby affirming their influence on consumer propensity to buy products derived from GM techniques. Indeed, people with higher education have a more negative view of GM products. In addition, among the women who reported that they receive information about food products, the higher their level of education, the lower their propensity to purchase GM foods was. Results say that consumers are less likely to purchase GM food, while age groups are positively related to the propensity to buy GM foods. However, people over 60 have a non-significant coefficient. The results seem to claim that the people who are most affected on the awareness of benefits and risks are the young ones. Italian women are more in favor of GM crops than men. The effect of income on the propensity to buy GM food is insignificant, even when we use the variable income classes.^b^^b^Results are not reported, but available upon request.

Given that the results are consistently reaffirmed, we can assert that the analysis remains robust.

Subsequently, we included a more specific detail on the knowledge of techniques by distinguishing them in three categories based on different biotechnological approaches: *random techniques* (induced gene mutation, crossing, and selection); *new techniques* (GMO and NBTs); and finally, *borderline techniques* (assisted selection with molecular markers, in vitro culture techniques), which cannot be classified as random or new as they utilize both classical approaches of crossing and selection and precise biotechnological techniques. The *borderline techniques* are the only ones reporting a significant and positive coefficient. This result could be explained by consumers knowing these methods and their purpose (efficient method for crop improvement/improved plant architecture). The results are presented in Appendix A.

The output of margins is in Tables 5, 6 and Table 7. Marginal effects show the change in probability when the independent variable increases by one unit. They give the difference in probability of each of the outcome levels associated with a unit change in each predictor variable.

**Table 5.** Margins from MNL regression (food safety).

	1: Not at all			2: I don’t know			3: Yes at all		
	dy/dx	SE		dy/dx	SE		dy/dx	SE	
Subj_knowl	0.054	0.026	**	−0.035	0.034		−0.019	0.024	
info_toll	0.006	0.018		−0.044	0.024	*	0.038	0.019	**
Obj_knowl	0.017	0.009	**	−0.003	0.012		−0.014	0.010	
Food_sust	−0.296	0.015	***	0.008	0.018		0.288	0.016	***
Africa_po	−0.036	0.044		−0.018	0.051		0.054	0.036	
Asian_po	−0.084	0.043	**	0.041	0.049		0.043	0.034	
American_po	−0.017	0.034		−0.107	0.043	**	0.124	0.035	***
European_po	−0.141	0.031	***	0.081	0.043	**	0.061	0.038	
Female	−0.081	0.028	***	0.008	0.036		0.073	0.030	**
Family_m	−0.010	0.018		0.004	0.022		0.006	0.018	
Marital_s	−0.009	0.030		0.017	0.039		−0.009	0.031	
Income	−0.034	0.020	*	0.024	0.025		0.010	0.021	
2.Age_c	−0.113	0.045	**	−0.015	0.057		0.128	0.043	***
3.Age_c	−0.092	0.045	**	0.020	0.056		0.072	0.044	
4.Age_c	−0.123	0.050	**	0.022	0.062		0.100	0.050	**
5.Age_c	0.016	0.052		0.038	0.072		−0.054	0.063	
2. degree	0.123	0.048	***	−0.115	0.057	**	−0.008	0.050	
3.degree	0.149	0.053	**	−0.117	0.064	**	−0.033	0.056	
4.degree	0.242	0.088	**	−0.166	0.105		−0.076	0.079	
2.area_geo	0.027	0.040		−0.081	0.053		0.054	0.044	
3.area_geo	0.012	0.043		−0.035	0.052		0.022	0.044	
4.area_geo	0.036	0.034		−0.098	0.043	**	0.063	0.036	*

**Table 6.** Margins from MNL regression (environmental sustainability).

	1: Not at all			2: I don’t know			3: Yes at all		
	dy/dx	SE		dy/dx	SE		dy/dx	SE	
Subj_knowl	0.025	0.012	**	−0.019	0.015		−0.006	0.013	
Obj_knowl	0.018	0.010	**	−0.014	0.012		−0.004	0.011	
Info_tool	0.030	0.021		−0.056	0.025	**	0.027	0.021	
Env_sust	−0.200	0.015	***	−0.013	0.024		0.212	0.022	***
African_po	−0.057	0.052		−0.042	0.055		0.100	0.041	**
Asian_po	−0.121	0.050	**	0.056	0.053		0.065	0.040	
American_po	−0.071	0.039	*	−0.085	0.045	**	0.156	0.038	***
European_po	−0.142	0.036	***	0.064	0.047		0.078	0.044	*
Femal	−0.052	0.032		0.002	0.038		0.050	0.033	
Family_n	0.007	0.020		−0.009	0.024		0.002	0.021	
Marital_s	−0.001	0.034		0.025	0.041		−0.024	0.036	
Income	−0.006	0.023		0.021	0.027		−0.015	0.024	
2.age_c	−0.122	0.051	**	−0.053	0.060		0.175	0.050	***
3.age_c	−0.101	0.050	**	−0.004	0.059		0.105	0.051	**
4.age_c	−0.107	0.055	***	−0.044	0.064		0.151	0.056	**
5.age_c	−0.019	0.060		0.017	0.076		0.002	0.073	
2.degree	0.071	0.055		−0.090	0.059		0.019	0.055	
3.degree	0.113	0.061	**	−0.113	0.067	*	0.001	0.061	
4.degree	0.067	0.094		−0.149	0.108		0.081	0.089	
2.area_go	0.025	0.046		−0.088	0.056		0.063	0.050	
3.area_geo	−0.028	0.048		−0.046	0.054		0.074	0.049	
4.area_geo	0.011	0.040		−0.106	0.046	*	0.095	0.041	**

**Table 7.** Margins from MNL regression (total benefit).

	1: Not at all			2: I don’t know			3: Yes at all		
	dy/dx	SE		dy/dx	SE		dy/dx	SE	
Subj_knowl	0.030	0.010	**	−0.026	0.014	**	−0.004	0.011	
Obj_know	0.016	0.009	*	−0.008	0.012		−0.008	0.010	
Info_tool	0.024	0.018		−0.045	0.024	**	0.021	0.019	
total_benefit	−0.154	0.008	***	−0.011	0.011		0.165	0.010	***
African_po	−0.027	0.045		−0.030	0.052		0.057	0.037	
Asian_po	−0.105	0.044	**	0.053	0.050		0.052	0.035	
American_po	−0.036	0.035		−0.078	0.044	*	0.115	0.035	***
Euroepan_po	−0.099	0.032	**	0.065	0.045		0.034	0.040	
Female	−0.073	0.029	***	−0.004	0.037		0.078	0.030	**
Family_m	0.001	0.018		0.002	0.023		−0.003	0.018	
Marital_s	−0.004	0.030		0.018	0.039		−0.014	0.031	
Income	−0.016	0.020		0.017	0.025	*	−0.001	0.021	
2.age_c	−0.127	0.045	**	−0.024	0.057		0.151	0.043	***
3.age_c	−0.123	0.045	**	0.028	0.056		0.094	0.043	**
4.age_c	−0.139	0.049	**	0.011	0.062		0.128	0.050	***
5.age_c	−0.036	0.052		0.061	0.072		−0.025	0.063	
2.degree	0.081	0.046	*	−0.083	0.057		0.002	0.049	
3.degree	0.105	0.052	**	−0.090	0.065		−0.015	0.054	
4.degree	0.148	0.077	**	−0.157	0.102		0.010	0.080	
2.area_go	0.025	0.040		−0.094	0.054	*	0.069	0.044	
3.area_geo	0.001	0.042		−0.045	0.052		0.044	0.043	
4.area_geo	0.022	0.035		−0.107	0.044	**	0.084	0.036	**

After adjustment for other explanatory variables in the “Not at all” group, a consumer having subjective knowledge on GM techniques increases the probability of propensity to purchase by 5.4 points. The analysis also shows that objective knowledge increases the probability of propensity to purchase by 1.7 points. The variable food sustainability is negative, and this result suggests a decrease in the propensity to buy equal to 29.6 by indicating a considerable sensitivity to the food sustainability issues. Product origin displays a negative coefficient for all the areas considered; however, only Asian and European product origins report a significant coefficient. Surprisingly, the dummy female is negative and significant, which could mean a persistence in the prior belief. Consumer behavior (buy) does not change between the different age classes; indeed, the coefficient is negative and statistically significant. It is interesting to highlight the sign of margins of the educational degree that is positive and significant, showing an increasing probability of purchasing GM food in the more educated group. Finally, the income level and probability of purchase are negatively related.

Moving to the results in the “I don’t know,” the beta coefficient of the information tool is negative and statistically significant. Therefore, the number of information tools utilized negatively impacts the decision to buy GM Food. Among the hesitant, a greater trust in European products than American ones is reported. The results associated with the educational degree are interesting; having a higher level of education increases the uncertainty of buying. Compared to the other regions, people living in the southern area, including the islands, are more reluctant to buy GM food.

In the analysis of the “Yes at all group,” the subjective and objective knowledge do not play a key role in determining the propensity to purchase, while the number of information tools does. Awareness of food sustainability issues drives the decision to buy GM food; if food sustainability increases by 1 unit, the propensity to purchase rises by 2.8. Product origin guides the propensity to buy in the case of North and South America. Conversely, concerning the “Not at all” group, the dummy female is positive and significant in the “Yes at all” group, indicating a higher confidence in female beliefs.

Age classes also change the sign in this group, showing a positive relation with the decision to buy, particularly in adults. Finally, southern areas converge toward a positive attitude.

These results are consistent with the ones of the other two models where the food sustainability variable was replaced by environmental sustainability and total benefit (sum of food and environmental sustainability perception). It is essential to underline that the size of the marginal effects of food sustainability is greater than one of the other variables, suggesting a more considerable sensitivity of consumers to food matters rather than environmental factors.

Moreover, we run the Hosmer-Lemeshow tests for goodness of fit, and the non-significant p-value signifies that there is no evidence that the observed and expected frequencies differ, which means evidence of a good fit.

### 3.1. Discussion

Regarding the first hypothesis, we find no evidence suggesting that objective and subjective knowledge is not aligned. Our analysis shows that knowledge drives a consumer’s choice to buy GM products in the “Not at all” group, where a higher level of knowledge positively impacts the propensity to buy these products. Our hypothesis fails as the two knowledge variables follow the same direction. Consumers who are already willing to buy GM food products do not need to increase their knowledge. In contrast, those in the “Not at all” group with a strong aversion toward these products could benefit from greater knowledge and a higher level of information. Substantially, increasing knowledge level, both real and perceived, would boost propensity to buy, even for strong opponents. This finding is in line with some parts of the literature,^9,25,26^ while diverging from the conclusions reached in a recent study.^27^

Concerning the second hypothesis, we differentiate the models as follows: in the first, we included a food safety variable but not environmental safety ones; in the second model, conversely, we included environmental safety but not food safety; in the third model, we included the total benefit resulting from the sum of both.

In the first model, the beta coefficients for food safety are significant and negative in the “Not at all” group, while they are positive in the “Yes at all” group. In the second model, previous results are also confirmed, but the beta coefficients are smaller. The same occurs in the third model as well. The results indicate that consumers strongly opposed to these products still do not buy GM products even if they perceive them as safer. We observe the same behavior in the “I don’t know” group, whereas, among consumers that are willing to buy, the relation between the food sustainability variable and the propensity to buy variable is positive. This is also true for environmental sustainability and total benefit. This indicates that these variables have the same influence on the propensity to buy, albeit to a different degree. More specifically, the size of the marginal effects suggests that consumers are more sensitive to the health-related component compared to environmental ones when discussing genetic modification.

Finally, concerning the third hypothesis, in the group of consumers who doubt purchasing GM products, the increase in information tools utilized decreases the propensity to buy, confirming a certain degree of distortion in the quality of communication and information about these products. While in the group of consumers that are already convinced to buy, adding tools of information positively affects the purchase decision. Therefore, our hypothesis is partially confirmed as we find that an unclear level of information about GMs affects consumers who are not convinced to buy, not giving them adequate tools to make an informed decision.

### 3.2. Robustness

We used an LR and Wald test to investigate whether specific variables have effects, either singly or jointly for each independent variable. Both tests lead to similar conclusions: the impact of educational degree, total benefits, a product of origin (Europe and North and South America), and female variables are significant. Therefore, rejecting the hypothesis that these variables do not affect the value considered important for the propension to buy (Table 8). The “Hausman tests of IIA assumption” seems to confirm that IIA has not been violated.

**Table 8.** LR TEST.

Variables	chi2	df	P>chi2
Subj_knowl	4.117	2	0.128
Obj_know	1.647	2	0.439
Info_tool	3.724	2	0.155
total_benefit	339.187	2	0.000
African_po	2.09	2	0.352
Asian_po	3.401	2	0.183
American_po	9.586	2	0.008
Euroepan_po	10.342	2	0.006
Female	10.285	2	0.006
Family_m	1.118	2	0.572
Marital_s	0.839	2	0.658
Income	0.353	2	0.409
2.degree	5.836	2	0.054
3.degree	5.349	2	0.069
4.degree	2.04	2	0.361
2.age	1.063	2	0.588
3.age	0.68	2	0.712
4.age	2.861	2	0.239

A confusion matrix (Table 9), accuracy test, and error rate (ER) have been computed to validate the classification model. The confusion matrix is a tool that helps us understand a classifier’s accuracy by comparing its output with the true values. It shows us the classification errors. The elements on the diagonal represent the number of correctly classified instances, while the elements outside the diagonal represent the number of misclassified ones.

**Table 9.** Mlogit confusion matrix structure.

		Predict		
		A	B	C
Actual	A	TRUE	FALSE	FALSE
	B	FALSE	TRUE	FALSE
	C	FALSE	FALSE	TRUE

When evaluating the performance of a binary classifier, the following terms are often used: TP for True Positive, FN for False Negative, TN for True Negative, and FP for False Positive. TP is the number of instances where the classifier correctly predicts the positive class as positive. TN is the number of cases where the classifier correctly predicts the negative class as negative. FN refers to the cases where the classifier incorrectly predicts the positive class as negative, which means that the data for those classes is rejected. To calculate FN, it is necessary to add up the values in the corresponding row, excluding the TP values. Finally, FP refers to the instances where the classifier incorrectly identifies negative values as positive. To calculate FP, adding up the values in the corresponding column, excluding the TP values is necessary.

The performance of a model is often measured by the accuracy of classification (ACC) and error rate. ACC represents the probability of correctly classifying a data point and is calculated using the formula: ACC = TP/(TP+TN+FP+FP). The error rate is the percentage of misclassified data points. In the consumption model, the overall accuracy is 75.70%, which indicates a higher probability of correctly classifying data points with an error rate of 24.30% (Table 10).

**Table 10.** Confusion matrix.

	predicted choice						
propensity to purchase		0	1	2	tot	FN	Overall FN
	0	147	27	9	183	36	137
	1	30	97	39	166	69	
	2	5	27	183	215	32	
	tot	**182**	**151**	**231**	**564**		
	FP	35	54	48			
	Overall TP	427					
	Overall FP	137					

**Figure 2.**
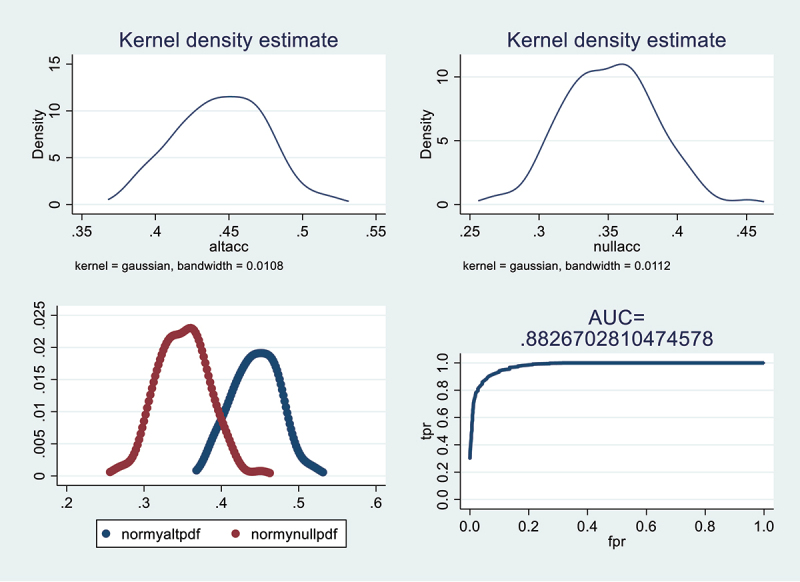
ROC Curve.

To evaluate how well the model predicts results, two key measures, sensitivity and specificity, are examined using a receiver operating characteristic (ROC) curve. Additionally, the area under the curve (AUC) is used to determine the quality of prediction. The figure displays the probability distributions for 100 alternative and null accuracy values using a technique called Kernel Density Estimation (KDE) with a Gaussian kernel. The false positive rate (FPR), true positive rate (TPR), and AUC are derived from the smooth probability density functions generated through KDE.^31^ The proposed consumption model performs well, with a high prediction rate of 88.26% (Figure 2).

## 4. Conclusion

The new applications and increasing adoption of biotechnology in agriculture target optimization of production factors by limiting inputs and resources. The empirical evidence has demonstrated that such technologies are at least as safe as traditional breeding technologies. Innovations based on these new research techniques provide substantial economic benefits and environmental improvements in critical ecosystems with overall important contributions toward sustainable development.^10^ Notably, our study fits into the recent scientific debate on this topic. On the one hand, we find that the prior belief holds in hesitant consumers, regardless of the information sources. On the other hand, consumers who are strongly adverse to GM products are willing to purchase these products if well informed, while at the opposite extreme, additional information on GM products does not have an impact on consumers who are already convinced to buy. Our results are in line with most of the empirical literature that emphasize the role of information in consumer’s choices.^18,22,32^ Of particular interest is the study of Bearth et al.^21^ which marks the importance of information that needs to be validated by scientific proofs to affect consumer’s purchasing behavior. In our case as well, information acts in different ways in the three different groups of consumers, suggesting the role of quality in information as well as consumer’s ability to understand the information itself.

We conclude that there is a need for more effective and transparent communication coming from trustworthy sources (e.g. European institutions, research centers, scientific journals) to speed up the process of acceptance of GM products. Finally, we are aware of the limits of this study concerning the construction of the objective knowledge variable, the length of the survey and the formulation of some questions. Nevertheless, this research enriches the current debate on consumer’s perception of GM food.

## Supplementary Material

Supplemental Material

## Data Availability

The authors will make the raw data supporting this article’s conclusions available without undue reservation.

## References

[1] Van Dijk M, Morley T, Rau ML, Saghai Y. A meta-analysis of projected global food demand and population at risk of hunger for the period 2010–2050. Nat Food. 2021;2(7):494–501. doi:10.1038/s43016-021-00322-9. 37117684

[2] Anders S, Cowling W, Pareek A, Gupta KJ, Singla-Pareek SL, Foyer CH. Gaining acceptance of novel plant breeding technologies. Trends Plant Sci. 2021;26(6):575–87. 33893048 10.1016/j.tplants.2021.03.004

[3] Dhankher OP, Foyer CH. Climate resilient crops for improving global food security and safety. Plant Cell Environ. 2018;41(5):877–84.Special Issue: Special Issue on Climate Resilient Crops. doi:10.1111/pce.13207. 29663504

[4] Demaria F, Zezza A. Scientific information and cognitive bias in the case of new breeding techniques: exploring millennials behaviour in Italy. Italian Rev Agric Econ. 2022;77(2):41–60. doi:10.36253/rea-13676.

[5] Ferrari L, Baum CM, Banterle A, De Steur H. Attitude and labelling preferences towards gene-edited food: a consumer study amongst millennials and generation Z. Br Food J. 2021;123(3):1268–86. doi:10.1108/BFJ-09-2020-0820.

[6] Ferrari L. Farmers’ attitude toward CRISPR/Cas9: the case of blast resistant rice. Agribusiness. 2022;38(1):175–94.

[7] Lassoued R, Smyth SJ, Phillips PW, Hesseln H. Regulatory uncertainty around new breeding techniques. Front Plant Sci. 2018;9:1291. doi:10.3389/fpls.2018.01291. 30233627 PMC6131982

[8] Noleppa and Cartsburg. The socio-economic and environmental values of plant breeding in the EU and for selected EU member states. Berlin, Germany: Hffa research paper; 2021.

[9] Sendhil R, Nyika J, Yadav S, Mackolil JG, Workie E, Ragupathy R, Ramasundaram P. Genetically modified foods: bibliometric analysis on consumer perception and preference. GM Crops Food. 2022 Dec 31. 13(1):65–85. doi:10.1080/21645698.2022.2038525. PMID: 35400312; PMCID: PMC9009926. 35400312 PMC9009926

[10] Smith V, Wesseler JHH, Zilberman D. New plant breeding technologies: an assessment of the political economy of the regulatory environment and implications for sustainability. Sustainability. 2021;13(7):3687. doi:10.3390/su13073687.

[11] European Commission. The European green deal (COM(2019) 640 final). Brussels, Belgium: European Commission. 2019. https://eur-lex.europa.eu/legal-content/EN/TXT/?uri=CELEX:52019DC0640.

[12] European Commission Communication COM/2020/381. Communication from the Commission to the European Parliament, the Council, the European Economic and Social Committee and the Committee of the Regions: a farm to fork strategy for a fair, healthy and environmentally-friendly food system. 2020.

[13] European Commission. Commission staff working document. Study on the status of new genomic techniques under union law and in light of the court of justice ruling in case C-528/16. Brussels, 29 4.2021 SWD(2021) 92 final. 2021.

[14] European Commission. Proposal for a regulation of the European Parliament and of the council on plants obtained by certain new genomic techniques and their food and feed, and amending regulation (EU) 2017/625. 2023.

[15] Mehta-Bhatt P, Ebora R, Cohen J, Falck-Zepeda J, Zambrano P. An overview of regulation, perceptions and priorities for GM crops in Asian countries. Asian Biotechnol Devel Rev. 2005;7(3):9–24.

[16] Bain C, Lindberg S, Selfa T. Emerging sociotechnical imaginaries for gene-edited crops for foods in the United States: implications for governance. Agric Hum Values. 2020;37(2):265–79. doi:10.1007/s10460-019-09980-9.

[17] Bunge J, Dockser MA. Is this tomato engineered? Inside the coming battle over gene-edited food. Wall Str J. 2018 Apr. 15. https://www.wsj.com/articles/is-this-tomato-engineered-inside-the-coming-battle-over-gene-edited-food-1523814992.

[18] Caputo V, Lusk JL, Kilders V. Consumer acceptance of gene edited foods: a nationwide survey on US consumer beliefs, knowledge, understanding and willingness to pay for gene-edited foods under different information treatments. Arlington (VA): FMI Foundation; 2020.

[19] Qaim M. Role of new plant breeding technologies for food security and sustainable agricultural development. Appl Econ Perspect Policy. 2020;42(2):129–50. doi:10.1002/aepp.13044.

[20] Sheldon IM. Regulation of biotechnology: will we ever ‘freely’trade GMOs? Eur Rev Agric Econ. 2002;29(1):155–76. doi:10.1093/erae/29.1.155.

[21] Bearth A, Drummond Otten C, Segrè Cohen A. Consumers’ perceptions and acceptance of genome editing in agriculture: insights from the United States of America and Switzerland. Food Res Int. 2024;178:113982. ISSN 0963-9969, 10.1016/j.foodres.2024.113982. 38309884

[22] Beghin JC, Gustafson CR. Consumer valuation of and attitudes towards novel foods produced with new plant engineering techniques: a review. Sustainability. 2021;13(20):11348. doi:10.3390/su132011348.

[23] Lusk JL, McFadden BR, Rickard BJ. Which biotech foods are most acceptable to the public? Biotechnol J. 2015;10(1):13–16. doi:10.1002/biot.201400561. 25388815

[24] Wuepper D, Wree P, Ardali G. Does information change German consumers’ attitudes about genetically modified food?. Eur Rev Agric Econ. 2019;46(1):53–78. doi:10.1093/erae/jby018.

[25] Hwang H, Nam S-J. The influence of consumers’ knowledge on their responses to genetically modified foods. GM Crops & Food. 2021;12(1):146–57. doi:10.1080/21645698.2020.1840911. 33138666 PMC7644159

[26] Wunderlich S, Smoller M. Consumer awareness and knowledge about food sources and possible environmental impact. Int J EI. 2019;2(1):85–96. doi:10.2495/EI-V2-N1-85-96.

[27] Bearth A, Siegrist M. Are risk or benefit perceptions more important for public acceptance of innovative food technologies: a meta-analysis. Trends Food Sci & Technol. 2016;49:14–23. doi:10.1016/j.tifs.2016.01.003.

[28] Wolf MM, Bertolini P, Shikama I, Berger A. A comparison of attitudes toward food and biotechnology in the US, Japan, and Italy. J Food Distribution Res. 2012;43(1):103–12.

[29] Ishii T, Araki M. Consumer acceptance of food crops developed by genome editing. Plant Cell Rep. 2016;35(7):1507–1518. 2016–07. doi:10.1007/s00299-016-1974-2. 27038939

[30] Romeo Lironcurti S, Demaria F, D’Annolfo R, Sardone R. Consumer evaluations of and attitudes towards new genome editing techniques: an Italian case study. Agriculture. 2024;14(1):51. doi:10.3390/agriculture14010051.

[31] -Peterson LE. MLOGITROC: stata module to calculate multiclass ROC Curves and AUC from multinomial logistic regression, statistical software components S457181. Boston College Department of Economics; 2010. RePEc:boc:bocode:s457181.

[32] Marette S, Disdier A-C, Beghin JC. A comparison of EU and US consumers’ willingness to pay for gene-edited food: evidence from apples. Appetite. 2021;159:105064. doi:10.1016/j.appet.2020.105064. 33278548

