# Supplementary material for: Consumers’ response to genetically modified food: an Italian case study
Source: GM Crops Food. 2024 Nov 6;15(1):303–15. doi: 10.1080/21645698.2024.2417473 (PMC11545258; doi:10.1080/21645698.2024.2417473)
Supplement: Supplemental Material [file KGMC_A_2417473_SM7074.docx]

**Annex**

**Appendix A: Ethical Approval**

*Data protection at Appinio*

Data protection and the associated protection of privacy are of central importance to Appinio as a market research company. We are committed to our clients and panelists to ensure at all times that all data is protected in accordance with the legal requirements of the GDPR. For Appinio, data protection and transparency about how data is handled is a principle we follow throughout our product development, also known as Privacy by Design. As a market research company, we have a special position and responsibility with respect to data privacy, as we are always in the middle between clients and survey participants. Below, we list resources that are available to you as an Appinio customer to inform you about our privacy practices. At the same time, this information will help you to also act in a privacy compliant manner and to ensure that your own privacy as well as that of the survey participants is protected at all times.

*Data minimisation and data retention*

Part of our data protection concept is data economy. According to our data retention policy, we only store the data we really need and only for as long as necessary. Data that users delete on Appinio will be removed within a maximum of 30 days. The only exceptions to this are very specific data, such as invoices and log files, which we need to keep in order to comply with our own legal obligations. For more information, please see our privacy policy.

*Data Security*

Encryption: All sensitive data (this includes all customer data, panelist data, and survey/response data) are secured via Sha265 encryption. All servers are also SSL secured, which means all data transfer is encrypted. Server location: All servers are located in Germany (Frankfurt). Appinio uses the infrastructure of AWS (Amazon Web Services) to guarantee the highest security standards and, at the same time, the highest accessibility of the systems. All survey data are stored here and made available exclusively to you, our customer, via our platform.

*Data usage and documents*

The following is information on the specific use of data that Appinio collects and provides. These data processing operations are also listed in our privacy policy and terms and conditions. All documents can be accessed in their original form at:

Customer T&Cs (for use of the survey platform) https://research.appinio.com/ /en/tos

Customer Privacy Policy (for website, platform, webapp) <https://www.appinio.com/en/privacy>

App Panelist T&Cs (accessible via app/play store and Appinio app) <https://link.appinio.com/#/en/tosApp>

Panelist Privacy Policy (accessible via app/play store and Appinio app) https://link.appinio.com/ /en/privacy

The app privacy policy lists what happens to our panelists’ data. The Website, Platform and Web App Privacy Policy lists how we handle data from our customers & web app participants. This information is intended to help understand and be transparent about the exact data processing procedures. Our privacy statements contain all the data processing provisions our customers need to be DSGVO compliant. In addition, our technical and organizational measures explain what security measures are in place internally to ensure data protection. After delivery of surveys, APPINIO collects the answers given by the end users and provides them in anonymous, aggregated form together with anonymous demographic and statistical data to the client in the form of an evaluation for market research purposes. Panelists provide us with this data voluntarily and to a self-determined extent. The data provided is aggregated and made available anonymously in a statistical evaluation that does not allow any conclusions to be drawn about individual users.

**Annex B: Questionnaire**

| **Question no.** | **Survey** | **Question type** |
| --- | --- | --- |
| **F1** | **What genetic improvement methods do you know? Please select all the methods you have heard about.**    A: Crossing and selection  B: Induced genetic mutation  C: Assisted selection with molecular markers  D: In vitro culture techniques  E: New breeding techniques (Nbts)  F: GMO  G: None of these (not randomized) | Multiple Choice  (Answers randomized) |
| **F2** | **Do you think there are differences between GMOs and products obtained through the use of New breeding techniques (Nbts)?**    A: Yes, there are differences and I know them  B: Yes, there are differences but I don’t know them  C: No, there are no differences  Q: I don’t know | Single Choice |
| **F3** | **For what purposes is genetic modification carried out? Select, from the following options, what you think are the purposes of genetic modification.**    A: Resistance to pests, diseases, herbicides  B: Adaptation to climate change  C: Reduce food waste/increase food yield by lowering production costs  Q: Reduce fertilizer use  E: Safeguarding biodiversity  F: I don’t know (not randomized) | Multiple Choice  (Answers randomized) |
| **F4** | **Based on your knowledge, which of these agricultural species is the most cultivated as GM crop?**    A: Soyabean  B: Corn  C: Wheat  D: Cotton  E: I don’t know (not randomized)  F: None of the above (not randomized) | Single Choice  (Answers randomized) |
| **F5** | **What information tools do you use to obtain information on GM products?**    A: Friends and acquaintances  B: Press and television  C: Social Media  D: Scientific and information publications  E: None of these (not randomized)  Other tools: (Freetext) | Multiple Choice  (Answers randomized) |
| **F6** | **Do you think GM food is safe to eat?**    A: Not at all safe  B: Not very safe  C: I don’t know  Q: Quite safe  E: Absolutely safe | Likert |
| **F7** | **In your opinion, could GM techniques contribute to environmental sustainability?**    A: Yes  B: No  C: I don’t know | Single Choice |
| **F8** | **How environmentally safe do you think GM techniques are?**    A: Not at all safe  B: Unsafe  C: I don’t know  Q: Quite safe  E: Absolutely safe | Likert |
| **F9** | **How likely is that you would buy a product derived from GM techniques?**    A: No chance  B: Low probability  C: I don’t know  Q: Some probability  E: High probability | Likert |
| **F10** | **From which region would you buy GM food?**    Answer:  A: yes  B: No  C: I don’t know    Items:  A: Africa  B: North America  C: Central and South America  D: Western Europe  E: Eastern Europe  F: Asia | Matrix  (Items randomized) |
| **F11** | **Which region do you live in?**    A: Aosta Valley  B: Piedmont  C: Liguria  D: Lombardy  E: Trentino- South Tirol  F: Veneto  G: Friuli Venezia Giulia  H: Emilia-Romagna  I: Tuscany  J: Umbria  K: Marche  L: Abruzzo  M: Lazio  N: Molise  O: Campania  P: Basilicata  Q: Apulia  R: Calabria  S: Sardinia  T: Sicily | Single Choice |
| **F12** | **Please indicate your educational level**    A: No qualification (primary school)  B: Secondary school  C: High school diploma  D: Bachelor’s degree  E: Master’s degree  F: Doctorate | Single Choice |
| **F13** | **What is your gross annual income?**    A: Less than 15,000 euros  B: Between 15,000 and 30,000 euros  C: Between 30,000 and 50,000 euros  Q: More than 50,000 euros | Single Choice |

**Appendix C: Regression results**

**Table C1**: MNL regression results

|  | (1) | (2) | (3) | (4) | (5) |
| --- | --- | --- | --- | --- | --- |
|  | est1 | est2 | est3 | est4 | est5 |
| **Base 1: Not al all** |  |  |  |  |  |
| 2 |  |  |  |  |  |
| Subj_knowl | **-0.21^**^** | **-0.21^**^** | **-0.32^***^** | **-0.34^***^** | **-0.31^***^** |
|  | (0.10) | (0.10) | (0.11) | (0.12) | (0.11) |
| Obj_knowl | **-0.15^*^** | **-0.15^*^** | -0.12 | -0.12 | -0.14 |
|  | (0.08) | (0.08) | (0.09) | (0.10) | (0.09) |
| Info_tool | **-0.31^*^** | **-0.33^*^** | -0.13 | -0.21 | -0.29 |
|  | (0.17) | (0.18) | (0.20) | (0.21) | (0.19) |
| Env_sust | **1.07^***^** | **1.07^***^** |  | **0.68^***^** |  |
|  | (0.16) | (0.16) |  | (0.19) |  |
| Africa_po | 0.15 | 0.15 | 0.23 | 0.19 | 0.17 |
|  | (0.43) | (0.43) | (0.40) | (0.41) | (0.43) |
| Asian_po | **0.84^**^** | **0.84^**^** | **0.57** | **0.73^*^** | **0.90^**^** |
|  | (0.39) | (0.40) | (0.40) | (0.41) | (0.42) |
| American_po | 0.24 | 0.24 | 0.07 | 0.14 | 0.22 |
|  | (0.30) | (0.30) | (0.35) | (0.37) | (0.35) |
| European_po | **0.93^***^** | **0.92^***^** | **1.19^***^** | **0.98^***^** | **0.87^***^** |
|  | (0.28) | (0.28) | (0.32) | (0.34) | (0.32) |
| Female | 0.30 | 0.30 | **0.57^**^** | **0.64^**^** | **0.55^*^** |
|  | (0.26) | (0.26) | (0.29) | (0.30) | (0.29) |
| Marital_s | 0.11 | 0.10 | 0.17 | 0.15 | 0.13 |
|  | (0.28) | (0.28) | (0.31) | (0.32) | (0.31) |
| Family_m | 0.01 | 0.01 | 0.16 | 0.15 | 0.10 |
|  | (0.12) | (0.12) | (0.13) | (0.14) | (0.13) |
| Degree | -0.28 | -0.29 | **-0.47^**^** | **-0.41^*^** | **-0.35^*^** |
|  | (0.20) | (0.20) | (0.21) | (0.21) | (0.20) |
| Income | 0.12 | 0.11 | **0.33^*^** | 0.28 | 0.20 |
|  | (0.17) | (0.17) | (0.20) | (0.20) | (0.19) |
| Age | 0.02 | 0.03 | 0.02 | 0.07 | 0.08 |
|  | (0.11) | (0.11) | (0.13) | (0.14) | (0.13) |
| Diff_methods |  | 0.09 | -0.10 | -0.05 |  |
|  |  | (0.27) | (0.32) | (0.32) |  |
| Food_sust |  |  | **2.17^***^** | **1.92^***^** |  |
|  |  |  | (0.27) | (0.29) |  |
| **Tot_benefit** |  |  |  |  | **1.15^***^** |
|  |  |  |  |  | (0.13) |
| _cons | -0.68 | -0.68 | -1.64^**^ | -2.20^**^ | -1.97^**^ |
|  | (0.72) | (0.72) | (0.81) | (0.87) | (0.84) |
| 3 |  |  |  |  |  |
| Subj_knowl | **-0.21^*^** | **-0.22^*^** | **-0.32^**^** | **-0.35^***^** | **-0.31^**^** |
|  | (0.12) | (0.12) | (0.13) | (0.13) | (0.13) |
| Obj_knowl | -0.14 | -0.15 | **-0.24^**^** | **-0.23^*^** | **-0.19^*^** |
|  | (0.09) | (0.10) | (0.12) | (0.12) | (0.11) |
| Info_tool | -0.01 | -0.04 | 0.21 | 0.08 | -0.02 |
|  | (0.17) | (0.18) | (0.24) | (0.24) | (0.21) |
| Env_sust | **2.16^***^** | **2.16^***^** |  | **1.24^***^** |  |
|  | (0.25) | (0.25) |  | (0.29) |  |
| African_po | **0.72^*^** | **0.73^*^** | 0.65 | 0.59 | 0.63 |
|  | (0.43) | (0.43) | (0.44) | (0.47) | (0.49) |
| Asian_po | **1.04^**^** | **1.03^**^** | **0.77^*^** | **1.00^**^** | **1.20^**^** |
|  | (0.42) | (0.42) | (0.44) | (0.46) | (0.49) |
| American_po | **1.31^***^** | **1.31^***^** | **1.24^***^** | **1.26^***^** | **1.25^***^** |
|  | (0.36) | (0.36) | (0.44) | (0.47) | (0.45) |
| Europena_po | **1.09^***^** | **1.09^***^** | **1.46^***^** | **1.16^***^** | **0.96^**^** |
|  | (0.36) | (0.36) | (0.41) | (0.44) | (0.43) |
| Female | **0.57^*^** | **0.56^*^** | **1.09^***^** | **1.26^***^** | **1.15^***^** |
|  | (0.30) | (0.30) | (0.36) | (0.39) | (0.38) |
| Marital_S | 0.01 | 0.01 | 0.17 | 0.14 | 0.09 |
|  | (0.31) | (0.31) | (0.37) | (0.38) | (0.36) |
| Family_m | -0.02 | -0.02 | 0.14 | 0.10 | 0.06 |
|  | (0.13) | (0.13) | (0.15) | (0.16) | (0.16) |
| Degree | -0.05 | -0.06 | **-0.47^*^** | -0.38 | -0.23 |
|  | (0.22) | (0.22) | (0.26) | (0.26) | (0.24) |
| Income | 0.06 | 0.04 | 0.35 | 0.30 | 0.21 |
|  | (0.21) | (0.21) | (0.24) | (0.25) | (0.24) |
| Age | 0.05 | 0.07 | -0.00 | 0.06 | 0.08 |
|  | (0.13) | (0.14) | (0.17) | (0.18) | (0.17) |
| Diff_methods |  | 0.21 | 0.18 | 0.19 |  |
|  |  | (0.33) | (0.39) | (0.41) |  |
| Food_sust |  |  | **4.19^***^** | **3.74^***^** |  |
|  |  |  | (0.40) | (0.40) |  |
|  |  |  |  |  |  |
| Total_benefit |  |  |  |  | **2.32^***^** |
|  |  |  |  |  | (0.24) |
| _cons | -4.66^***^ | -4.68^***^ | -6.38^***^ | -7.53^***^ | -7.49^***^ |
|  | (0.94) | (0.94) | (1.04) | (1.23) | (1.24) |
| *N* | 564 | 564 | 564 | 564 | 564 |
| adj. *R*^2^ |  |  |  |  |  |
| AIC | 969.39 | 972.95 | 818.01 | 797.81 | 821.40 |
| GOF | 0.949 | 0.944 | 0.979 | 0.000 | 0.006 |

Notes: Reference group = None at all; −2Log likelihood = - 312.19677; Chi-square = 191.43, N.obs 564, Standard errors in parentheses significance level * p < 0.10, ** p < 0.05, *** p < 0.01, geographical area fixed effects are included in the regressions

**Table C2:** MNL regression

|  | (1) | (2) | (3) |
| --- | --- | --- | --- |
|  | est1 | est2 | est3 |
| Base 1 Not at all |  |  |  |
| 2 |  |  |  |
| Subj_knowl | **-0.19^*^** | **-0.33^***^** | **-0.30^***^** |
|  | (0.11) | (0.11) | (0.11) |
| Obj_knowl | **-0.14^*^** | -0.13 | -0.14 |
|  | (0.08) | (0.09) | (0.09) |
| Info_tool | **-0.32^*^** | -0.13 | -0.28 |
|  | (0.17) | (0.19) | (0.19) |
| Env_sust | **1.10^***^** |  |  |
|  | (0.16) |  |  |
| Afrinc_po | 0.22 | 0.22 | 0.17 |
|  | (0.45) | (0.44) | (0.48) |
| Asian_po | **0.83^**^** | **0.72^*^** | **0.96^**^** |
|  | (0.41) | (0.43) | (0.44) |
| America_po | 0.18 | -0.06 | 0.13 |
|  | (0.31) | (0.35) | (0.37) |
| Europena_po | **0.98^***^** | **1.33^***^** | **0.94^***^** |
|  | (0.29) | (0.33) | (0.34) |
| Female | **0.30** | **0.67^**^** | **0.59^**^** |
|  | (0.26) | (0.29) | (0.30) |
| Marital_s | 0.07 | 0.06 | 0.05 |
|  | (0.28) | (0.31) | (0.32) |
| Family_m | -0.02 | 0.09 | 0.03 |
|  | (0.12) | (0.13) | (0.13) |
| Income | 0.09 | 0.33 | 0.17 |
|  | (0.17) | (0.20) | (0.19) |
| 2.degree | -0.65 | **-1.23^**^** | **-0.83^*^** |
|  | (0.44) | (0.48) | (0.48) |
| 3.degree | **-0.95^*^** | **-1.44^***^** | **-1.04^*^** |
|  | (0.50) | (0.55) | (0.55) |
| 4.degree | -0.77 | **-2.31^**^** | **-1.51^**^** |
|  | (0.84) | (0.94) | (0.77) |
| 2.age_c | 0.55 | **0.91^*^** | **0.99^**^** |
|  | (0.43) | (0.51) | (0.49) |
| 3.age_c | 0.56 | **0.90^**^** | **1.05^**^** |
|  | (0.41) | (0.44) | (0.50) |
| 4.age_c | 0.49 | **1.13^**^** | **1.15^**^** |
|  | (0.44) | (0.50) | (0.52) |
| 5.age_c | 0.16 | 0.04 | 0.42 |
|  | (0.47) | (0.55) | (0.57) |
| Food_sust |  | **2.43^***^** |  |
|  |  | (0.30) |  |
| Total_benefit |  |  | **1.24^***^** |
|  |  |  | (0.14) |
| _cons | -0.89 | -2.15^***^ | -2.35^***^ |
|  | (0.69) | (0.78) | (0.81) |
| 3 |  |  |  |
| Subj_knowl | -0.17 | **-0.29^**^** | **-0.28^**^** |
|  | (0.12) | (0.13) | (0.13) |
| Obj_knowl | -0.12 | -**0.23^**^** | -0.19 |
|  | (0.10) | (0.12) | (0.12) |
| Info_tool | -0.03 | 0.24 | -0.04 |
|  | (0.18) | (0.23) | (0.22) |
| Env_sust | **2.24^***^** |  |  |
|  | (0.27) |  |  |
| African_po | **0.83^*^** | 0.63 | 0.63 |
|  | (0.47) | (0.49) | (0.54) |
| Asian_po | **1.03^**^** | **0.96^**^** | **1.24^**^** |
|  | (0.44) | (0.47) | (0.51) |
| American_po | **1.22^***^** | **1.08^**^** | **1.14^**^** |
|  | (0.38) | (0.46) | (0.48) |
| European_po | **1.22^***^** | **1.61^***^** | **1.07^**^** |
|  | (0.39) | (0.45) | (0.47) |
| Female | 0.56^*^ | **1.18^***^** | **1.18^***^** |
|  | (0.30) | (0.37) | (0.39) |
| Marital_s | -0.09 | 0.01 | -0.04 |
|  | (0.32) | (0.38) | (0.38) |
| Family_m | -0.06 | 0.04 | -0.05 |
|  | (0.13) | (0.15) | (0.15) |
| Income | -0.04 | 0.36 | 0.13 |
|  | (0.22) | (0.25) | (0.25) |
| 2.degree | -0.30 | -1.03 | -0.63 |
|  | (0.52) | (0.67) | (0.63) |
| 3.degree | -0.64 | **-1.39^*^** | -0.97 |
|  | (0.59) | (0.74) | (0.71) |
| 4.degree | 0.04 | -2.49^**^ | -1.12 |
|  | (0.84) | (1.06) | (0.84) |
| 2.age_c | **1.60^***^** | **1.89^***^** | **2.15^***^** |
|  | (0.52) | (0.60) | (0.60) |
| 3.age_c | **1.12^**^** | **1.41^***^** | **1.68^***^** |
|  | (0.50) | (0.52) | (0.60) |
| 4.age_c | **1.38^**^** | **1.85^***^** | **2.06^***^** |
|  | (0.54) | (0.63) | (0.68) |
| 5.age_c | 0.10 | -0.41 | 0.10 |
|  | (0.69) | (0.81) | (0.84) |
| Food_sust |  | **4.51^***^** |  |
|  |  | (0.44) |  |
| Total_benefit |  |  | **2.47^***^** |
|  |  |  | (0.27) |
| _cons | -5.16^***^ | -7.58^***^ | -8.45^***^ |
|  | (0.97) | (1.21) | (1.38) |
| *N* | 564 | 564 | 564 |
| adj. *R*^2^ |  |  |  |
| AIC | 968.59 | 806.47 | 814.02 |
| GOF | 0.482 | 0.238 | 0.000 |

Standard errors in parentheses significance level ^*^ *p* < 0.10, ^**^ *p* < 0.05, ^***^ *p* < 0.01; geographical fixed effects are included in the regressions.

**Table C3:** MNL regressions

|  | (1) | (2) | (3) |
| --- | --- | --- | --- |
|  | est1 | est2 | est3 |
| Base 1: Not at all |  |  |  |
| 2 |  |  |  |
| Subl_knowl | -0.41 | **-0.60^**^** | **-0.61^**^** |
|  | (0.26) | (0.28) | (0.28) |
| Kn_random | 0.63 | 0.53 | 0.63 |
|  | (0.43) | (0.52) | (0.50) |
| K_border | 0.63 | 0.76 | **0.93^*^** |
|  | (0.46) | (0.49) | (0.51) |
| K_new | -0.44 | -0.21 | -0.34 |
|  | (0.41) | (0.45) | (0.45) |
| Obj_knwol | **-0.14^*^** | -0.14 | -0.15 |
|  | (0.08) | (0.09) | (0.09) |
| Info_tool | **-0.33**^*^ | -0.15 | -0.30 |
|  | (0.18) | (0.19) | (0.19) |
| Env_sust | **1.12^***^** |  |  |
|  | (0.16) |  |  |
| African_po | 0.24 | 0.25 | 0.22 |
|  | (0.46) | (0.45) | (0.49) |
| Asian_po | **0.79^**^** | **0.78^*^** | **0.97^**^** |
|  | (0.40) | (0.43) | (0.45) |
| American_po | 0.22 | -0.06 | 0.16 |
|  | (0.31) | (0.35) | (0.37) |
| European_po | **0.96^***^** | **1.31^***^** | **0.92^***^** |
|  | (0.29) | (0.33) | (0.33) |
| Female | 0.29 | **0.65^**^** | **0.59^*^** |
|  | (0.26) | (0.29) | (0.30) |
| Marital_s | 0.11 | 0.07 | 0.09 |
|  | (0.29) | (0.32) | (0.33) |
| Family_m | -0.02 | 0.10 | 0.03 |
|  | (0.13) | (0.13) | (0.14) |
| Income | 0.12 | **0.36^*^** | 0.21 |
|  | (0.18) | (0.20) | (0.20) |
| 2.degree | -0.57 | **-1.21^**^** | -0.76 |
|  | (0.45) | (0.48) | (0.49) |
| 3.degree | **-0.86^*^** | **-1.35^**^** | **-0.95^*^** |
|  | (0.50) | (0.55) | (0.56) |
| 4.degree | -0.48 | **-2.17^**^** | -1.26 |
|  | (0.88) | (0.93) | (0.79) |
| 2.age_c | 0.47 | 0.82 | **0.87^*^** |
|  | (0.45) | (0.50) | (0.49) |
| 3.age_c | 0.54 | **0.83^*^** | **0.97^*^** |
|  | (0.44) | (0.45) | (0.53) |
| 4.age_c | 0.38 | **1.01^**^** | **0.96^*^** |
|  | (0.46) | (0.51) | (0.53) |
| 5.age_c | 0.08 | -0.06 | 0.28 |
|  | (0.49) | (0.55) | (0.58) |
| Food_sust |  | **2.44^***^** |  |
|  |  | (0.31) |  |
| Total_benefit |  |  | **1.26^***^** |
|  |  |  | (0.14) |
| _cons | -0.89 | -2.13^***^ | -2.39^***^ |
|  | (0.69) | (0.74) | (0.79) |
| 3 |  |  |  |
| Subj_knowl | **-0.53^*^** | **-0.63^**^** | **-0.68^**^** |
|  | (0.30) | (0.31) | (0.32) |
| K_random | 0.55 | 0.15 | 0.28 |
|  | (0.48) | (0.57) | (0.59) |
| K_border | **1.02^*^** | **1.26^**^** | **1.43^**^** |
|  | (0.53) | (0.59) | (0.62) |
| K_new | -0.04 | 0.00 | -0.01 |
|  | (0.48) | (0.54) | (0.56) |
| Obj_knowl | -0.12 | **-0.23^**^** | -0.18 |
|  | (0.10) | (0.12) | (0.12) |
| Info_tool | -0.03 | 0.25 | -0.05 |
|  | (0.18) | (0.23) | (0.22) |
| Env_sust | **2.28^***^** |  |  |
|  | (0.27) |  |  |
| African_po | **0.89^*^** | 0.71 | 0.72 |
|  | (0.48) | (0.49) | (0.56) |
| Asian_po | **1.02^**^** | **0.99^**^** | **1.28^**^** |
|  | (0.43) | (0.47) | (0.51) |
| American_po | **1.28^***^** | **1.14^**^** | **1.22^**^** |
|  | (0.38) | (0.46) | (0.48) |
| European_po | **1.16^***^** | **1.54^***^** | **0.98^**^** |
|  | (0.39) | (0.44) | (0.46) |
| Female | **0.54^*^** | **1.12^***^** | **1.14^***^** |
|  | (0.31) | (0.38) | (0.39) |
| Marital_s | -0.06 | 0.01 | -0.01 |
|  | (0.33) | (0.39) | (0.39) |
| Family_m | -0.08 | 0.04 | -0.06 |
|  | (0.13) | (0.15) | (0.16) |
| Income | -0.03 | 0.36 | 0.15 |
|  | (0.22) | (0.26) | (0.26) |
| 2.degree | -0.23 | -1.00 | -0.56 |
|  | (0.51) | (0.69) | (0.66) |
| 3.degree | -0.59 | **-1.29^*^** | -0.88 |
|  | (0.58) | (0.77) | (0.74) |
| 4.degree | 0.31 | **-2.35^**^** | -0.87 |
|  | (0.89) | (1.07) | (0.86) |
| 2.age_c | **1.48^***^** | **1.73^***^** | **1.97^***^** |
|  | (0.54) | (0.60) | (0.62) |
| 3.age_c | **1.02^*^** | **1.27^**^** | **1.53^**^** |
|  | (0.53) | (0.54) | (0.64) |
| 4.age_c | **1.21^**^** | **1.65^***^** | **1.79^**^** |
|  | (0.57) | (0.64) | (0.70) |
| 5.age_c | -0.03 | -0.57 | -0.10 |
|  | (0.70) | (0.82) | (0.85) |
| Food_sust |  | **4.61^***^** |  |
|  |  | (0.45) |  |
| Total_benefit |  |  | **2.55^***^** |
|  |  |  | (0.27) |
| _cons | -5.18^***^ | -7.65^***^ | -8.56^***^ |
|  | (0.97) | (1.20) | (1.37) |
| *N* | 564 | 564 | 564 |
| adj. *R*^2^ |  |  |  |
| AIC | 969.53 | 808.41 | 813.22 |
| GOF | 0.626 | 0.240 | 0.000 |
